# The MuRQoL-He—Hebrew Adaptation of the Music Related Quality of Life Questionnaire Among Adults Who Are Deaf and Hard of Hearing

**DOI:** 10.3390/audiolres15050127

**Published:** 2025-09-28

**Authors:** Zahi Tubul, Zvi Tubul-Lavy, Gila Tubul-Lavy

**Affiliations:** 1 Hearing Institute and Cochlear Implant Program, Kaplan Medical Center, Rehovot 76100, Israel; 2 Virtual Worlds R&D, F-Narrative Venture, Holon 5867905, Israel; zvitubul@gmail.com; 3 Department of Communication Sciences and Disorders, Ono Academic College, Kiryat Ono 55000, Israel; gilatu@gmail.com

**Keywords:** music perception, quality of life, hearing loss, cochlear implants, hearing aids, patient-reported outcomes, auditory rehabilitation

## Abstract

**Purpose:** The present study aimed to describe the adaptation and validation process of the MuRQoL (Music Related Quality of Life questionnaire) from English to Hebrew and to describe normative data from a cohort of adults with normal hearing versus those with hearing aids or cochlear implants. **Methods**: After thoroughly translating and adapting to Hebrew, the participants completed the questionnaire online. We calculated the Cronbach’s alpha and McDonald’s omega scores for all scales and subscales. The construct validity of the questionnaire was evaluated using Confirmatory Factor Analysis (CFA) and the “known group” method. A total of 310 adults participated in this study. Fifty-four participants were deaf or hard of hearing, and 256 had normal hearing. **Results**: Internal consistency of the MuRQoL-He scales and subscales demonstrated good-to-excellent reliability. The goodness-of-fit indices for the frequency and importance scales were within acceptable standards. We found a significant difference in the frequency scale, where the normal-hearing group scores were significantly higher than those of the deaf and hard-of-hearing groups. **Conclusions:** The validity and reliability of the MuRQoL-He have been confirmed, indicating that it is suitable for guiding music rehabilitation for Hebrew-speaking deaf and hard-of-hearing adults.

## 1. Introduction

Hearing loss (HL) is a widespread sensory impairment affecting individuals of all ages and demographics. The consequences of hearing loss extend beyond the loss of auditory perception and affect an individual’s communication abilities, social interactions, and overall quality of life [1,2]. Managing hearing loss often involves a multidisciplinary approach, including one or more of the following: hearing aids, cochlear implants, assistive listening devices, and auditory rehabilitation programs. Early detection and intervention are crucial for mitigating the harmful effects of hearing loss, emphasizing the importance of regular audiology screenings and public health initiatives to promote hearing health awareness [3,4].

### 1.1. Music and Quality of Life

Music has been integral to human culture for centuries, transcending language barriers and fostering emotional expression and communication. Numerous studies have suggested a strong correlation between music and its positive impact on overall quality of life. The activation of reward pathways in the brain by listening to music has been linked to enhanced emotional regulation and overall psychological well-being [5]. Moreover, active music-making, such as singing or playing an instrument, improves self-esteem, mood, and life satisfaction [6]. Engaging with music has demonstrated positive effects on cognitive functions such as memory, attention, and executive skills. Regular participation in music activities has been linked to improved verbal memory and language processing [7].

Recent studies have shown that musical training can enhance auditory perception and cognitive abilities, leading to better speech perception under noisy conditions and enhanced working memory performance [8,9]. Moreover, music has been suggested to positively impact mental health and well-being, including reducing stress and anxiety and improving mood and quality of life [6,10].

### 1.2. Music and Hearing Loss

Music perception in individuals with hearing impairment presents unique challenges and variations compared with those with normal hearing (NH). Frequency resolution abilities may partially explain the variability in hearing aid users’ perceptions of music quality [11]. Strategies such as auditory training, assistive devices, and visual cues have been explored to enhance the musical experiences of individuals with hearing impairments [12,13]. Furthermore, technological advancements, such as cochlear implants and assistive listening devices, have expanded opportunities for deaf and hard-of-hearing (DHoH) individuals to participate in musical experiences [14]. Research has demonstrated that individuals with DHoH can still derive emotional and social benefits from music through residual hearing and other sensory modalities [15]. Understanding the multifaceted relationship between music and quality of life can inform future interventions and support programs for the general population and communities with hearing impairment.

### 1.3. Existing Questionnaires

Several questionnaires have been developed to evaluate music perception and enjoyment after cochlear implantation. One commonly used tool is the University of Washington Clinical Assessment of Music Perception Test (UW-CAMP), which assesses various aspects of music perception in cochlear implant users [16]. Another questionnaire is the Dutch Musical Background Questionnaire (DMBQ), which measures music listening habits, quality of sound, and self-assessed perception of elements of music [17]. The Iowa Music Perception and Appraisal Battery, Primary Measures of Music Audiation Test Battery, and Music Excerpt Recognition Test are frequently used to assess music perception in cochlear implant users. These questionnaires provide insights into music perception and enjoyment, including melody recognition, rhythm perception, and overall music appreciation [18,19]. The Iowa Musical Background Questionnaire (IMBQ) is a self-report questionnaire that assesses the quality of music perception through a cochlear implant and the factors that may influence listening to music, such as musical training and habits [20]. These questionnaires are valuable tools for evaluating the impact of cochlear implantation on music perception and enjoyment, and they contribute to a better understanding of the experiences of cochlear implant users of music. However, the existing questionnaires do not consider the influence of musical perception and music consumption habits on a person’s quality of life implications or the importance individuals attribute to music experiences for rehabilitation purposes.

### 1.4. The MuRQoL

The Music-related Quality of Life Questionnaire (MuRQoL) is a multidimensional assessment tool designed by [21] to measure the impact of music on individuals’ quality of life. The MuRQoL questionnaire demonstrates strong reliability and validity as a self-report tool for assessing music perception, engagement, and importance in adults using cochlear implants. Perception refers to the subjective ability to detect and recognize musical features; Importance refers to the personal value attributed to music in daily life; and Engagement refers to the frequency and intensity of participation in musical activities. Its potential lies in guiding music aural rehabilitation strategies to enhance the music experiences of cochlear implant users. The MuRQoL questionnaire comprises two sets of 18 items each: one evaluates music experiences, and the other gauges their significance. These items have content and face validity, and each set was evaluated using a consistent 5-point Likert scale [21]. To illustrate, items include the following: ‘Can you follow the melody in music (i.e., the melody of a familiar tune)?’ (perception), ‘How important is it for you to be able to recognise the words in songs?’ (importance), ‘Do you attend public music events (e.g., musicals, concerts, or music festivals)?’ (engagement), and ‘Do you usually listen to music whilst travelling (e.g., in the car)?’ (frequency). The MuRQoL questionnaire addressed the limitations of previous music questionnaires intended for adult cochlear implant (CI) users. It can bridge this gap by offering a dependable and valid means of assessing the outcomes of music-centered interventions. Additionally, the MuRQoL questionnaire could serve as a screening tool in clinical settings by assessing how music influences an individual’s quality of life, thus helping identify specific rehabilitation needs. The MuRQoL can also help clinicians identify which aspects of music (e.g., perception vs. engagement) are most affected for each individual. This information can guide personalized rehabilitation strategies such as music-based auditory training or counseling. The MuRQoL has been translated into Turkish [22], Italian [23], and Spanish [24]. The questionnaire was found to be valid and reliable, cross-culturally applicable, and of significant clinical value.

### 1.5. The Need for a Hebrew Version of Self-Reported Music-Related QoL

Estimates suggest that there are nearly 15 million Hebrew-speaking people worldwide, most in Israel. Israel is a state of 22,000 square kilometers in the Middle East with a population of 10 million. As of 2015, 13% of Israel’s population above the age of 20 reported difficulty hearing, comprising 680,000 people. The deaf community in Israel is estimated to include more than 7000 people [25].

To our knowledge, no validated questionnaire in Hebrew measures quality of life in the musical context and considers the importance of the ability to perceive music and participate in musical activities for individuals with hearing loss. Patients and clinicians might use such a tool to identify personal needs when building an intervention plan for optimal hearing rehabilitation and to monitor a person’s progress in the rehabilitative aspect.

### 1.6. Aims of the Present Work

A. To describe the adaptation of the MuRQoL to Hebrew and the validation process of the Music Related Quality of Life questionnaire (Hebrew).
B. To present normative data from a cohort of adults with normal hearing and those with hearing aids or cochlear implants.

## 2. Materials and Methods

### 2.1. Translation of the Questionnaire

The translation was conducted according to the guide for translating and adapting hearing-related questionnaires for different languages and cultures [26] and additional suggestions [27]. In light of these recommendations, the process of translation and adaptation to Hebrew was carried out while adhering to each of the proposed steps. Our study was conducted following ethical standards and was approved by the institutional ethics committee of the Ono Academic College (approval number: 202472-ono). After receiving permission from the author of the original tool for use and translation, MuRQoL was translated into Hebrew by two independent bilingual (English and Hebrew) certified translators. The two translated versions were then compared to the original tool, and the differences between them were discussed and resolved using a committee approach by the researchers and a third bilingual certified translator. The preliminary Hebrew version was then back-translated into English by a fourth independent translator, and arguments were again resolved using a committee approach. The pre-final version was sent to five healthcare professionals and five DHoH individuals as a pilot for the next step. All participants marked this version as clear and understood. Minor changes were made after the investigators and the fourth translator reached a complete agreement. The final version of the MuRQoL-He used in the present work is available from the corresponding author upon request.

### 2.2. Participants

Normal hearing (NH) and people who are deaf and hard of hearing (DHoH) adults participated in this study. The inclusion criterion for all participants was an age ≥ 18 years. The NH participants self-reported no history of diagnosed hearing loss, no complaints of hearing difficulties, and no use of hearing devices, with no ear or hearing complaints or problems. For all participants, the demographic information included age, sex, marital status, level of education, religious definition, and mean income. DHoH participants were also asked to upload their latest unaided audiogram performed at a certified audiology clinic by a certified audiologist. The authors ranked the participants hearing loss severity according to each ear’s unaided pure-tone average (PTA) of 0.5, 1, 2 & 4 kilohertz by the following index: 0–15 dB-normal range, 16–25 dB-slight decline, 26–40 dB-mild, 41–55 dB-moderate, 56–70 dB-moderately severe, 71–90 dB-severe, and above 91 dB for profound hearing loss. Only subjects with moderate hearing loss in one or more of their ears were included in the DHoH group to ensure the DHoH group represented clinically significant cases. DHoH participants also declared the type of hearing instrument they used (unilateral or bilateral hearing aid, cochlear implant, or no instrument used), hearing loss onset, etiology of hearing loss, and time since they were fitted with their first instrument (hearing aid or cochlear implant).

All participants were recruited through the Internet and social media and completed the questionnaire and demographic questions online via the Google Forms platform. Data were collected from 19 July 2023, to 7 March 2024. Before completing the questionnaires, each participant confirmed informed consent to participate in the study after receiving all the necessary information according to the accepted ethics and standards of responsible conduct of research. All participants completed the MuRQoL-He, comprising 18 frequency items measured on a 5-item scale ranging from “Never” to “Always”. These items were categorized into two subscales: assessing music perception and evaluating music engagement. In addition, 18 corresponding importance items were measured on a 5-item scale from “Not important at all” to “Extremely important”. The Not Applicable (N/A) option was also available throughout the questionnaire. All 36 items from the original MuRQoL were retained in the Hebrew version without modifications. Finally, the subjects were asked one question about the degree of comprehensibility and clarity of the questionnaire measured on a 4-item scale from “Not clear at all” to “Very clear”.

### 2.3. Tools

In the MuRQoL questionnaire, we used 36 items from the original scale, translated into Hebrew. This included 11 items each for music perception and its corresponding importance and seven for music engagement and its importance. We prepared two versions of the questionnaire: one for the NH group and the other for the DHoH group. Responses were rated on a Likert scale from 1 (never) to 5 (always). A “Not Applicable” (NA) option was also available. If a participant selected the NA option, we calculated their score by excluding irrelevant items. Most participants in both groups (82.6%) did not select this option. Less than 3% chose it more than twice among the 36 items, and none of the participants chose it more than five times.

Consequently, we opted not to exclude participants who chose the NA option. The Mann–Whitney test indicated no significant differences between the groups in selecting the NA option (*Z* = −1.725, *p* = 0.084). We chose this test over the *t*-test because of the significant differences in the group sizes.

### 2.4. Statistical Approach

To assess the internal reliability of the questionnaire, we calculated Cronbach’s alpha for each of the four subscales and two major scales: frequency and importance. Additionally, we computed McDonald’s omega [28], as suggested in recent studies [29,30,31]. Scholars have reached a growing consensus on using McDonald’s omega over Cronbach’s alpha. Unlike alpha, omega is based on structural equation modeling techniques, explicitly utilizing confirmatory factor analysis (CFA) loadings. A key advantage of omega over alpha is its consideration of the strength of the relationships between items and constructs and item-specific measurement errors. Thus, omega offers a more accurate estimate of scale reliability than does Cronbach’s alpha. For both measures, a score above 0.7 is typically considered acceptable, above 0.8 is good, and above 0.9 is excellent. We calculated alpha and omega scores using the psych package in R [32].

Following the procedure of previous validation efforts of the Italian, Spanish, and Turkish versions [22,23,24], the construct validity of the questionnaire was evaluated using Confirmatory Factor Analysis (CFA). Construct validity was further assessed using the known group method, a psychometric approach that evaluates whether a tool can differentiate between groups expected to vary on the construct under study (e.g., NH vs. DHoH participants). This provides evidence that the MuRQoL-He is sensitive to clinically meaningful group differences [26,28]. Goodness-of-fit indices served as independent evaluation criteria for the CFA results. The most popular fit indices in the literature include the Comparative Fit Index (CFI), Tucker–Lewis’s index (TLI), Root Mean Square Error of Approximation (RMSEA), and standardized Root Mean Square Residual (SRMR). There is a vast body of literature concerning acceptable values. A general rule of thumb suggests that RMSEA and SRMR should be lower than 1, preferably lower than 0.8, and CFI and TLI should be above 0.9, preferably above 0.95 [33]. Exploratory factor analysis (EFA) and CFA were executed using The Jamovi open-source software version 2.2.5 [34].

The “known group” method assessed the questionnaire’s construct validity by comparing the scores across the NH and DHoH groups. A scale is deemed valid if it yields significantly different scores for groups known to differ in a particular concept. Following the Italian validation effort [23], we employed the non-parametric Mann–Whitney U test to compare the groups. Furthermore, a non-parametric test was more appropriate because group sizes differed significantly. Additionally, given the differences between the NH and DHoH groups in mean age, sex, and education level, we employed multiple linear regressions to examine whether the groups differed on various scales and subscales when controlling for these demographic variables.

In addition, following previous validation efforts, EFA was conducted separately for the 18 frequency and 18 importance items to identify the underlying factors based on their relationships. We employed the principal axis factoring extraction method with a varimax rotation. The Kaiser–Meyer–Olkin (KMO) measure and Bartlett’s test of sphericity were used to assess the significance of the factor structures. A KMO value near one suggests adequate sampling, while items with factor loadings below 0.30 should be considered for removal. A *p*-value < 0.05 from the Bartlett test confirms sampling adequacy [35].

### 2.5. Participants’ Characteristics

A total of 310 adults were included in the final analysis, comprising 256 participants with normal hearing (NH) and 54 participants who were deaf or hard of hearing (DHoH). Table 1 summarizes the sociodemographic and clinical characteristics of both groups. In general, participants in the DHoH group were older on average, included a higher proportion of males, and presented heterogeneous profiles in terms of hearing loss severity, onset, etiology, and device usage. Notably, most DHoH participants had severe-to-profound bilateral hearing loss, with approximately half reporting prelingual onset. Almost all DHoH participants used hearing devices, most commonly cochlear implants or bimodal fittings. These characteristics provide important context for interpreting group differences in the subsequent analyses.

## 3. Results

Table 2 presents the reliability scores of the scales and subscales of the entire sample. This table also includes the mean score on each (sub) subscale and standard deviations.

The reliability scores found in the current study were slightly smaller than those found in the English (original) and Turkish versions [22,23]; however, they were all within the acceptable range, and most were considered good or excellent. Notably, these reliability scores were similar to those found in the Italian and Spanish versions of MuRQoL [23,24]. Following the procedure in previous validation efforts [22,23], we examined the goodness-of-fit indices obtained from the CFA for both the frequency and importance scales.

As shown in Table 3, the indices were either acceptable or slightly below the acceptable standards. The RMSEA score for the frequency scale was 0.084, and the importance scale was 0.116 (0.05 ≤ RMSEA ≤ 0.10 indicates acceptable fit). The SRMR was within normal limits for both scales: 0.063 for frequency and 0.094 for importance (0.05 ≤ SRMR ≤ 0.10 indicates acceptable fit). However, the CFI and TLI scores were lower than acceptable, particularly for the importance scale (Table 3). The goodness-of-fit indices were slightly lower than those found in the Turkish version [22] and similar to those in the Italian and Spanish versions [23,24].

**Figure 1:**
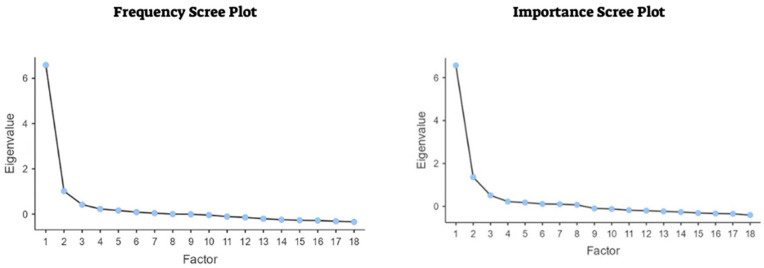
Scree plots of exploratory factor analysis of the frequency and importance scales of MuRQoL-He.

As mentioned above, we extracted two factors or subscales for the frequency and importance scales named in previous research: music perception and engagement. It is worth noting that some items had factor loadings below 0.30 in our EFA. Nevertheless, we extracted two factors for each scale to maintain consistency with the previous studies [22,23,24]. It is also worth mentioning that the Turkish and Italian versions had a few items with factor loadings below 0.30. Figure 1 presents the scree plots for the frequency and importance scales. Factor retention was guided by visual inspection of scree plots, which display the eigenvalues associated with successive factors. The ‘elbow’ of the plot, where the curve begins to flatten, was used in combination with the Kaiser criterion (eigenvalues > 1.0) to determine the optimal number of factors. Both methods supported a double factor solution, consistent with previous adaptations of the MuRQoL. Table 4 and Table 5 present the results for the EFAs. Factor loadings represent the correlation of each item with the latent factor. Uniqueness refers to the proportion of variance in an item not explained by the extracted factors (i.e., 1—communality).

The obtained dimensions showed structural significance based on the Bartlett test results (for the frequency scale: χ^2^ = 2013, *p* < 0.001; for the importance scale: χ^2^ = 2464, *p* < 0.001). The overall KMO for the frequency scale was 0.918, and for the importance scale, it was 0.902. These statistics indicate that the variances of the two factors, perception and engagement, differ. Perception explained 22.7% of the frequency scale, whereas engagement explained 20.5%. Together, these two factors accounted for 43.2% of the frequency scale. For the importance scale, the perception factor accounted for 24.2%, and the engagement factor accounted for 21.5%, totaling 45.7%. Although some items (e.g., 4, 7, and 10) demonstrated relatively low factor loadings, none of them showed substantially higher loadings on the alternative factor. For the sake of comparability with previous adaptations of the MuRQoL, all items were retained in the final solution.

Next, we used the “known group” method to assess the questionnaire’s construct validity by comparing scores across the two distinct groups: NH and DHoH. The results of the Mann–Whitney non-parametric tests are presented in Table 6.

As Table 6 shows, we found significant differences between the groups for all the scales and subscales. An exception was the engagement factor of the importance scale, for which no significant differences emerged. These data further confirm the reliability of the questionnaire as they indicate that it can detect differences between hearing and hearing-impaired groups.

We also conducted multiple linear regressions to examine whether the group variable (NH vs. DHoH) could predict the frequency and importance of the two outcome variables beyond demographic data. We were particularly interested in the effects of the group variable while controlling for age, as there were age differences between the groups, with older participants in the DHoH group compared to those in the NH group (see Section 2.2). Additionally, we controlled for sex and educational level. Table 7 presents the results of these two regression models.

The outcome variable in the first model (Table 7, top panel) is the frequency. The model was statistically significant, *F* (4, 305) = 41.696, *p* < 0.001, explaining 35.4% of the variance. The table shows that the group variable was the strongest predictor of frequency. The interpretation of the results indicates that the NH group scored, on average, 0.873 points higher than the DHoH group on a 1–5 scale, controlling for demographics. Furthermore, it is evident from the table that younger participants and females scored higher on the frequency scale.

The outcome variable is influential in the second model (Table 7, bottom panel). The model was statistically significant, *F* (4, 305) = 3.950, *p* = 0.004, explaining 4.9% of the variance. Table 7 shows that the group variable was, again, the strongest predictor of frequency. The interpretation of the results indicates that the DHoH group scored, on average, 0.248 points higher than the NH group on a 1–5 scale, controlling for demographics. Furthermore, it is evident from the table that females scored higher on the importance scale.

The NH group scored higher on the frequency scale, whereas the DHoH group scored higher on the importance scale, suggesting an interactive effect. We employed a two-way mixed-model ANCOVA to examine this interaction, controlling for age, sex, and education level. The between-group variable was “group” (NH/DHoH), and the within-group variable was “scale” (frequency/importance). As hypothesized, an interaction effect emerged, *F* (1, 305) = 137.346, *p* < 0.001, with a very strong effect size, η_p_^2^ = 0.310 [36]. The analysis also indicated the main effects for the group, *F* (1, 305) = 15.275, *p* < 0.001, η_p_^2^ = 0.048, and scale, *F* (1, 305) = 8.450, *p* = 0.004, η_p_^2^ = 0.027.

**Figure 2:**
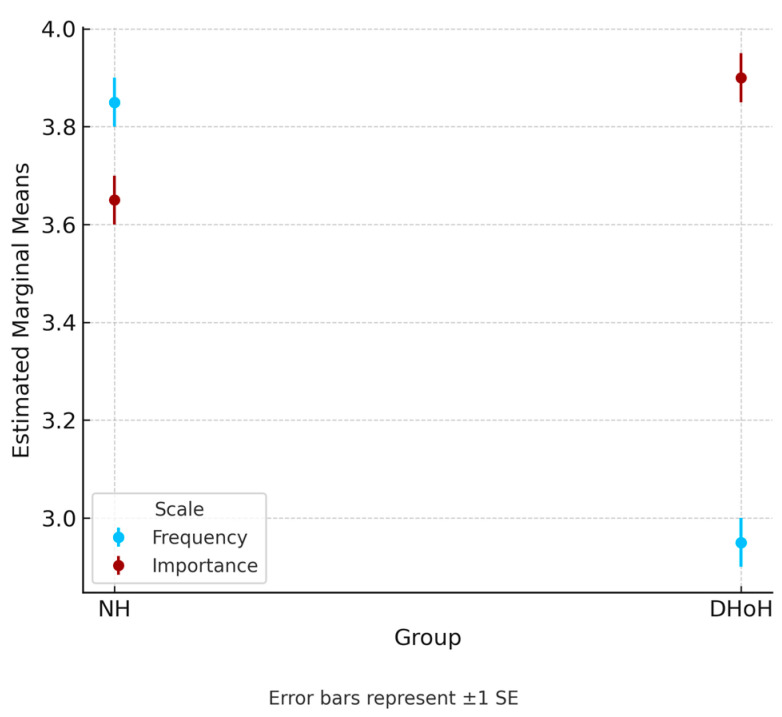
Marginal means of NH and DHoH groups on the frequency and importance scales.

To interpret this interaction, we plotted the results (Figure 2). From the figure, it is evident that while there is a significant difference in the frequency scale, where the NH group scores are significantly higher than those of the DHoH group, the differences are more modest in the importance scale and the opposite direction, with the DHoH group having slightly higher scores than the NH group.

## 4. Discussion

Music perception and participation in music activities among cochlear implant users are crucial for enhancing their quality of life. Despite the challenges in perceiving music owing to limitations in pitch and timbre cues, studies have shown that music training can positively impact the recognition and enjoyment of music in this population [37,38]. Assessing music perception is essential because it has been linked to improved self-reported hearing ability and overall quality of life [17]. Understanding and measuring music perception in this group sheds light on their auditory capabilities and is vital for enhancing their well-being. Therefore, the present study’s first aim was to describe the adaptation of MuRQoL to Hebrew and the validation process of MuRQoL-He.

The internal consistency of the MuRQoL-He scales and subscales was assessed using Cronbach’s α, which demonstrated good-to-excellent reliability. These results align with the values from the original study, indicating significantly high internal consistency within the questionnaire. The goodness-of-fit indices obtained from the CFA for both the frequency and importance scales were either acceptable or slightly below acceptable standards. The CFI and TLI scores were slightly lower than those found in the Turkish version [22] and similar to those in the Italian and Spanish versions [23,24]. This indicates that the overall model fit is acceptable, though not optimal. Such findings suggest that while the factorial structure is largely supported, certain items may not fully capture the underlying constructs in the Hebrew-speaking population. Therefore, the results should be interpreted with some caution, and future studies might consider confirmatory analyses with larger and more diverse samples to further refine the model.

We conclude that the validity and reliability of the MuRQoL-He have been confirmed, indicating that it is suitable for guiding music rehabilitation for Hebrew-speaking deaf and hard-of-hearing adults who use hearing aids or cochlear implants.

Second, we aimed to present normative data from a cohort of adults with normal hearing versus those with hearing aids or cochlear implants. It is evident that while there was a significant difference in the frequency scale, where the NH group scores were significantly higher than the DHoH group scores, the differences were more modest in the importance scale and the opposite direction, with the DHoH group having slightly higher scores than the NH group.

Our findings showed a statistically significant difference in the frequency scale scores between the two groups. This indicates that the questionnaire effectively assessed concepts relevant to individuals with hearing impairment and could discern these differences. Conversely, a modest statistically significant difference was found in the importance scale scores, suggesting that cochlear implant users value music as much as individuals with normal hearing or even more, despite facing significant challenges in perceiving it. This finding aligns with the existing literature, highlighting the significance of music for adult cochlear implant users, even in the absence of optimal music perception and enjoyment observed in previous studies [39,40].

The current study’s limitations can be related to sampling bias, as 50% of the DHoH group reported prelingual hearing loss, and to the participants’ recruitment by the Internet and social media, which may not reflect the whole population. As most participants were recruited online, the sample may be biased toward younger and more technologically engaged individuals. Therefore, future studies should recruit through hospitals and audiology clinics to enhance representativeness and generalizability. A second issue is the heterogeneity of the DHoH group, which included CI, HA, and bimodal users with varied etiologies and onset times. Due to the small subgroup sizes, the study was underpowered to examine outcome differences between device types, which limits conclusions about potential variability across CI and HA users and can affect the ability to generalize the research conclusions to all hearing-impaired adults. Future research should aim to recruit larger and more balanced subgroups to allow meaningful comparisons. An additional limitation of this study is that we did not assess test–retest reliability or sensitivity to change. Future longitudinal studies are needed to establish these psychometric properties of the MuRQoL-He.

Future research should also focus on assessing the effectiveness of music rehabilitation programs on the quality of life and music perception skills of Hebrew-speaking adult cochlear implant users. Additionally, investigating the relationship between music perception skills, quality of life, and participation in music activities among Hebrew-speaking cochlear implant users could provide valuable insights into the benefits of music rehabilitation tailored to this specific patient. Using the MuRQoL-He questionnaire, future research can compare the music-related quality of life among adults with hearing loss who use hearing devices versus those who do not to evaluate the contribution of hearing instruments to the individual’s music-related quality of life. This study introduced and validated the MuRQoL-He questionnaire, providing the first tool to assess the impact of music experience on well-being and music perception among Hebrew-speaking individuals. These findings support its potential for both clinical application and future research.

## Figures and Tables

**Table 1:** Sociodemographic and clinical characteristics of study participants.

Characteristic	NH (n = 256)	DHoH (n = 54)
N participants	256	54
Mean age (years, SD, range)	40.5 (13.6), 18–67	48.7 (17.5), 21–80
Gender (Female/Male, %)	73.4/26.6	63.0/37.0
Married (%)	70.3	61.1
University diploma (%)	83.6	77.7
Income (Average/Above average, %)	41.4/38.7	48.1/33.3
HL severity (severe–profound, %)	–	83.3 (left)/75.9 (right)
HL onset (Pre/Post/Perilingual, %)	–	50.0/38.9/11.1
HL etiology known (%)	–	76.0
Device use (%)	–	Bimodal 33.3; Bilateral CI 31.5; Bilateral HA 20.4; Unilateral HA 9.3; Unilateral CI 3.7
CI brand (n)	–	Cochlear 18; Advanced Bionics 14; MED-EL 4
Time since first device (years, SD, range)	–	9.9 (6.8), 1–29 (n = 37)

**Table 2:** Reliability and mean Scores (n = 310).

Scale	α	ω	Mean	SD
Frequency perception	0.893	0.897	3.94	0.66
Frequency engagement	0.733	0.786	3.33	0.71
Importance perception	0.890	0.893	3.70	0.77
Importance engagement	0.791	0.805	3.60	0.72
Frequency total	0.906	0.911	3.71	0.62
Importance total	0.907	0.910	3.67	0.70

Note: α = Cronbach’s alpha; ω = McDonald’s omega; SD = standard deviation.

**Table 3:** CFA Goodness-of-fit indices of the Hebrew version of the MuRQoL (n = 310).

Fit Indices	Good Fit	Acceptable Fit	Hebrew	
			Frequency Scale	Importance Scale
N	-	-	310	310
Df	-	-	134	134
χ^2^	0 ≤ χ^2^ ≤ 2 df	0 ≤ χ^2^ ≤ 3 df	431	693
*p*	0.05 ≤ *p* ≤ 1.00	0.001 ≤ *p* ≤ 0.05	<0.001	<0.001
χ^2^/df	0 ≤ χ^2^ ≤ 2 df	0 ≤ χ^2^ ≤ 3 df	3.216	5.172
SRMR	0.00 ≤ SRMR ≤ 0.05	0.05 ≤ SRMR ≤ 0.10	0.063	0.094
RMSEA	0.00 ≤ RMSEA ≤ 0.05	0.05 ≤ RMSEA ≤ 0.10	0.084	0.116
CFI	0.90 ≤ CFI ≤ 0.95	0.95 ≤ CFI ≤ 1.00	0.870	0.779
TLI	0.90 ≤ TLI ≤ 0.95	0.95 ≤ TLI ≤ 1.00	0.852	0.748

Note: Df = Degrees of Freedom; SRMR = Standardized Root Mean Square Residual; RMSEA = Root Mean Square Error of Approximation; CFI = Comparative Fit Index; TLI = Tucker–Lewis’s index.

**Table 4:** KMO measure of sampling adequacy and exploratory factor analysis for the frequency scale.

Scale	Item	KMO MSA	Factor Loading	Uniqueness
Perception	1	0.879	0.801	0.457
	2	0.893	0.708	0.414
	3	0.926	0.744	0.518
	4	0.923	0.231	0.603
	5	0.920	0.705	0.462
	6	0.935	0.524	0.613
	7	0.952	0.270	0.458
	8	0.906	0.398	0.542
	9	0.937	0.645	0.459
	10	0.939	0.252	0.568
	11	0.945	0.381	0.615
Engagement	12	0.916	0.614	0.613
	13	0.905	0.702	0.543
	14	0.908	0.685	0.582
	15	0.889	0.727	0.542
	16	0.933	0.485	0.663
	17	0.878	0.312	0.815
	18	0.931	0.673	0.752

Note: KMO MSA = Kaiser–Meyer–Olkin Measure of Sample Adequacy.

**Table 5:** KMO measure of sampling adequacy and exploratory factor analysis for the importance scale.

Scale	Item	KMO MSA	Factor Loading	Uniqueness
Perception	1	0.902	0.883	0.213
	2	0.879	0.778	0.344
	3	0.903	0.859	0.244
	4	0.878	0.226	0.747
	5	0.853	0.729	0.388
	6	0.906	0.420	0.649
	7	0.914	0.284	0.599
	8	0.945	0.454	0.487
	9	0.932	0.601	0.488
	10	0.904	0.335	0.699
	11	0.927	0.446	0.673
Engagement	12	0.904	0.690	0.510
	13	0.915	0.737	0.436
	14	0.897	0.617	0.594
	15	0.877	0.646	0.573
	16	0.851	0.484	0.633
	17	0.942	0.369	0.760
	18	0.897	0.223	0.740

Note: KMO MSA = Kaiser–Meyer–Olkin Measure of Sample Adequacy.

**Table 6:** Non-parametric Mann–Whitney tests comparing the NH and DHoH groups on the frequency and importance scales.

Measure	NH (n = 256)		DHoH (n = 54)		Mann–Whitney	
	Median	Range	Median	Range	*U*	*p*
Frequency perception	4.14	1.27–5.00	3.32	2.18–4.91	2490	<0.001
Frequency engagement	3.57	1.00–4.43	2.50	1.29–4.86	2451	<0.001
Importance perception	3.76	2.18–5.00	4.09	1.00–4.91	4947	0.001
Importance engagement	3.71	1.00–5.00	3.86	1.00–5.00	6795	0.845
Frequency total	3.89	1.17–4.78	2.97	2.06–4.83	2117	<0.001
Importance total	3.72	1.89–5.00	3.97	1.00–4.94	5658	0.036

Note: NH = Normal Hearing; DHoH = deaf and hard of hearing.

**Table 7:** Results of multiple regressions predicting frequency (top panel) and importance (bottom panel).

Frequency			
Model	*B*	*β*	*p*
Constant	3.992	-	<0.001
Group (NH = 0, DHoH = 1)	−0.873	−0.517	<0.001
Age	−0.005	−0.125	0.010
Gender (male = 0, female = 1)	0.209	0.147	0.002
Education level	−0.034	−0.054	0.256
Importance			
Model	*B*	*β*	*p*
Constant	3.846	-	<0.001
Group (NH = 0, DH = 1)	0.248	0.136	0.019
Age	−0.005	−0.105	0.072
Gender (male = 0, female = 1)	0.205	0.133	0.021
Education level	−0.074	−0.109	0.060

Note: *B* = unstandardized coefficient; *β* = standardized coefficient. Education level: 0 = high school graduate, 1 = professional diploma, 2 = BA, 3 = MA, 4 = doctoral); n = 309.

## Data Availability

Data supporting the findings of this study are contained within the article. Further inquiries can be directed to the corresponding author.
